# Practical Observations on Hysteria, Especially Relating to Its Organic Character

**Published:** 1838-10

**Authors:** 


Art. IV.—*Practical Observations on Hysteria, especially relating to its Organic Character.* By John Prichard, Member of the Royal College of Surgeons, London; and Surgeon to the Leamington Hospital.—*Leamington*, 1838. 8vo. pp. 36.

The object of this pamphlet appears to be that of showing that the nature and origin of hysteria are not so undefinable, and its treatment is not so unsatisfactory, as they are generally apprehended to be. Its author sets out by a suggestion, that it is "much oftener an organic affection than we are disposed to consider it;" and he at the same time expresses his conviction "that, in each and every case, it is to be regarded as emanating from uterine disturbance." He maintains that the disturbance may be very slight; but confidently assumes that, however slight it is, it is the cause of the hysteric phenomena. The irritation, he conceives, is transmitted to the brain, and from thence to other parts of the nervous system, "and especially to the par vagum and its important connexions." All cases of this kind he terms *cerebral* hysteria, in contradistinction to painful local affections, which he classes under the head of *neuralgic* hysteria: but he considers even these last to be generally occasioned by uterine disease or disturbance; the sympathy being in them *directly* communicated. The present publication chiefly relates to this latter form; and its principal object is to point out the proneness of such affections to pass on to the inflammatory state; particularly illustrating it by cases of long-continued pain, referrible to the situation of the cardiac orifice of the stomach, and terminating in perforating ulcer.

We confess we have some difficulty in referring all these cases to uterine irritation, and to classing them under the head of hysteria. But there are several excellent practical observations scattered through this little work. Various interesting points are touched upon in a manner which proves Mr. Prichard to be an observing and reflecting practitioner; and, if he had allowed his ideas a little more space, and been a little more careful in the arrangement of his subjects, a clearer impression would have been left on the reader's mind than the disquisition in its present form is calculated to produce.

